# A simple and reliable tool to quantify calcium burden of ascending aorta

**DOI:** 10.1186/1749-8090-8-S1-O20

**Published:** 2013-09-11

**Authors:** E Gatta, G Rescigno, V Polverini, MD Pierri, L Carbonari, A Giovagnoni, L Torracca

**Affiliations:** 1 Vascular Surgery Department, Ospedali Riuniti di Ancona, Ancona, Italy; 2 Cardiac Surgery Department, Ospedali Riuniti di Ancona, Ancona, Italy; 3 Radiology Department, Ospedali Riuniti di Ancona, Ancona, Italy

## Background

Ascending aorta calcific plaques represent a potential source of emboli during open-heart procedures. The aim of our study was to validate a simple and new technique to quantify aorta calcium burden.

## Methods

Ten thoracic CT scans (TCMD General Electric LightSpeed VCT 64 Slices) of octogenarian subjects were analysed by two radiologists and one vascular surgeon, using the Osirix Pro Software. A 3D reconstruction of the ascending thoracic aorta was obtained from the annular plane to the origin of the innominate trunk. The outer curvature maximal length was measured; therefore this value was divided by ten Region Of Interest (ROI) points. At each ROI an exact perpendicular section of the aorta was obtained, whose calcium involvement was expressed as a percentage. The overall calcium burden was expressed as the mean of the ten measurements.

## Results

Cronbach test estimate was 0.975. Intraclass correlation coefficient (ICC) was 0.927 (95% confidence limits = 0.904-0.946) with F test = 39.3 and p < 0.001.

## Conclusion

Our technique represents a simple and effective way to provide quantitative assessment of ascending aorta calcium burden. There is an excellent intra-observer congruity with high reproducibility of measurements. This method may be particularly useful when screening optimal candidates for transcatheter aortic valve implantation.

